# Impact of oral contraceptive use on muscle mass and strength in women with PCOS

**DOI:** 10.1007/s12020-025-04305-9

**Published:** 2025-06-13

**Authors:** Seren Aksun, Levend Karaçoban, Ilkay Idilman, Bulent O. Yildiz

**Affiliations:** 1 Hacettepe University School of Medicine, Department of Internal Medicine, Ankara, Turkey; 2 Hacettepe University School of Medicine, Department of Internal Medicine, Division of Endocrinology and Metabolism, Ankara, Turkey; 3 Hacettepe University School of Medicine, Department of Sports Medicine, Ankara, Turkey; 4 Hacettepe University School of Medicine, Department of Radiology, Ankara, Turkey

**Keywords:** Polycystic ovary syndrome, Oral contraceptive, Adiposity, Body composition, Insulin resistance, Muscle strength

## Abstract

**Purpose:**

Polycystic ovary syndrome (PCOS) is characterized by androgen excess and ovulatory dysfunction and appears to be associated with alterations in muscle mass and function. The study aims to investigate whether oral contraceptive (OC) use affects muscle mass and strength in women with PCOS.

**Methods:**

Twenty women with PCOS (median age 20.5 years and BMI 26.1 kg/m^2^) and 20 age- and BMI-matched healthy controls were included. Clinical, hormonal, and biochemical assessments were conducted along with body composition analyses using magnetic resonance imaging (MRI) proton density fat fraction (PDFF%) and muscular strength assessment by isokinetic dynamometry. In women with PCOS, measurements were repeated after at least three cycles of OC therapy.

**Results:**

At baseline, women with PCOS exhibited significantly higher levels of total testosterone, free androgen index (FAI), and homeostasis model assessment of insulin resistance (HOMA-IR) levels compared to healthy controls (*p* < 0.001, *p* = 0.001, *p* = 0.004, respectively). PCOS group also showed significantly higher average power (AvP) of knee extensors at 60°/sec (*p* = 0.002). AvP correlated positively with total testosterone and FAI levels in the whole study group (r = 0.450, *p* = 0.004, r = 0.318, *p* = 0.045, respectively). Following OC therapy, testosterone levels and FAI decreased (*p* = 0.02 and *p* < 0.001, respectively); whereas thigh muscle mass or lower limb strength remained unchanged.

**Conclusions:**

Short-term OC use in women with PCOS led to a reduction in androgen excess without measurable effects on muscle composition or strength. These findings suggest that muscle function and composition remain stable over the short term, despite hormonal modulation. Further research is required to understand how long-term management strategies for PCOS might affect muscle mass and function.

## Introduction

Polycystic ovary syndrome (PCOS) represents the most prevalent endocrine and metabolic disorder among women of reproductive age, characterized by clinical/biochemical hyperandrogenism (HA), oligo-anovulation (OA), and polycystic ovarian morphology (PCOM) [1, 2]. Androgen excess and insulin resistance are recognized as central components in the complex pathophysiology of PCOS [3]. These abnormalities not only contribute to reproductive and metabolic disturbances but also influence body composition.

Available data on muscle mass in women with PCOS are rather conflicting. While some studies report reduced or unchanged total lean mass compared to controls [4, 5], others demonstrate increased total and trunk muscle mass in women with PCOS [6, 7]. Recent evidence indicates that women with PCOS, regardless of BMI, often present with increased visceral adiposity and alterations in skeletal muscle composition, which are closely associated with insulin resistance and metabolic dysfunction [8, 9]. These changes in body composition may contribute to the reproductive and metabolic abnormalities and long-term cardiometabolic risk. In this context, advanced imaging modalities such as magnetic resonance imaging-proton density fat fraction (MRI-PDFF%) and magnetic resonance spectroscopy (MRS) enable non-invasive and accurate quantification of muscle fat infiltration and composition [10].

In women with PCOS, insulin resistance and hyperinsulinemia trigger a pathogenic cascade that enhances muscle anabolism and stimulates androgen excess [11, 12]. Elevated androgen levels further promote muscle mass and strength while exacerbating insulin resistance, thereby perpetuating a vicious metabolic cycle [13, 14]. The combined effects of insulin resistance and hyperandrogenism significantly disrupt muscle metabolism and contribute to systemic metabolic dysfunction [12]. Given this complex interplay between insulin, androgens, and muscle physiology in PCOS, objective assessment of muscle strength is essential. The isokinetic dynamometer is a reliable tool for assessing quadriceps and hamstring muscle strength, assessing both eccentric and concentric muscle force at set velocities [15]. The most commonly used measurements obtained from an isokinetic dynamometer are peak torque (PTQ), reflecting maximum explosive force and average power (AvP), representing force output over time [16]. Two recently published meta-analyses have reaffirmed the validity and reliability of PTQ and AvP measurements using isokinetic dynamometry for muscle strength evaluation [17, 18].

Oral contraceptives (OCs) are the first-line pharmacological agents for the management of PCOS, as they reduce gonadotropin secretion and ovarian androgen production and regulate the menstrual cycle [19]. This therapeutic approach increases levels of sex hormone-binding globulin (SHBG), which in turn lowers free testosterone concentration and mitigates hirsutism [20]. Although the metabolic and cardiovascular effects of OCs in women with PCOS have been well studied, their impact on musculoskeletal health remains unclear [21]. Suppression of endogenous androgens by OCs may influence muscle mass and strength, which are critical for metabolic health, physical performance, and quality of life [22]. Understanding these effects may contribute to more individualized treatment strategies in PCOS. Accordingly, this study aims to investigate the impact of OC use on muscle mass and strength, highlighting a less recognized aspect of the syndrome.

## Materials and methods

### Study population and clinical protocol

The study protocol was approved by the Ethics Committee of Hacettepe University (2019/04-44) and written informed consent was obtained from all participants.

The study included 20 treatment-naive patients with PCOS presented to the outpatient endocrinology and metabolism clinic of the Hacettepe University and 20 age- and BMI-matched healthy volunteers. PCOS diagnosis was established based on the Rotterdam criteria [23], as previously described [24]. Based on three components of the syndrome according to the Rotterdam criteria, (hyperandrogenism: HA, oligoanovulation: OA, and polycystic ovarian morphology: PCOM), PCOS is classified into four phenotypes: phenotypes A (HA + OA + PCOM), B (HA + OA), C (HA + PCOM) and D (OA + PCOM). This study included only patients with phenotypes A and B [25]. Participants were eligible for inclusion if they had a BMI between 18 and 35 kg/m^2^.

Clinical and laboratory assessments were performed on all individuals during the early follicular phase, days 2 through 5 of the menstrual cycle. For participants with oligomenorrhea, oral medroxyprogesterone acetate was administered to induce withdrawal bleeding. Demographic and anthropometric data, including height, weight, hip and waist circumferences, and blood pressure, were collected. Body mass index (BMI) and waist-to-hip ratio (WHR) were then calculated using the formulas: weight (kg) / height (m)², and waist (cm) / hip (cm) respectively. Blood samples for hormonal and metabolic assessments were obtained after at least 8 h of fasting, between 8:00 and 10:00 in the morning.

Following clinical and biochemical evaluation, muscle strength was measured by isokinetic dynamometry, and composition analyses were performed by MRI-PDFF %. All participants in the PCOS group received an OC, dienogest-etinylestradiol (2/0.03 mg) in accordance with the international guideline recommendations [26]. The measurements were repeated using the same methods during the early follicular phase in women with PCOS, after at least three cycles of OC use during the hormone-free interval.

### Laboratory measurements

Total testosterone, SHBG, fasting glucose and fasting insulin were measured as previously reported [27]. Total cholesterol, low-density lipoprotein (LDL), high-density lipoprotein (HDL) and triglyceride (TG) levels were measured by enzymatic method (Beckman Coulter, AU 5800 Clinical Chemistry Analyzers, USA).

Standard equations were used to calculate the free androgen index (FAI) and the homeostasis model assessment index-insulin resistance (HOMA-IR) values. (FAI = [Total testosterone (mmol/l) / SHBG (nmol/l) *100]), HOMA-IR = [fasting insulin ((U/ml) *fasting plasma glucose (FPG) (mg/dl) /405]).

Assessment of muscle composition and mechanical function analyses.

All patients underwent magnetic resonance imaging (MRI) with a 1.5-T system (Siemens AERA, Germany) with standard body and spine matrix coils for the assessment of body composition analyses. All MRI measurements were performed by using a workstation (syngo. via VB10; Siemens Medical Solutions). A single reader (I.I.) with seventeen years of experience verified all measures. The MRI-PDFF measurement technique was previously reported [9]. Additionally, in this study, the entire thigh is considered to extend from the proximal to the distal ends of the femur, and the middle third was selected for measuring the area and fat fraction of the thigh muscles. A freehand region of interest (ROI) was used for measurement of thigh muscle area by avoiding the femur and vessels on the opposed-phase image. The ROI was copied and pasted on the fat fraction image for measuring intramuscular fat.

The single, calibrated isokinetic dynamometer device Biodex® System 3 (Biodex Corp, Shirley, NY, USA) was used by the same sports physician (L.K.) at Hacettepe University, Department of Sports Medicine, to assess the muscle strength of each participant. Before muscle strength analyses, the participants performed a warm-up exercise on a treadmill (G-THORC TX4000. SAS SPOR, TR) for 10 min at a speed of 6 km/h. The details of the technique for measuring the strength of muscles were previously published [27]. The analyses results were obtained in the following units: total work in joules (J), average power (AvP) in watts (W), and extensor and flexor peak torque in Newton-meters (Nm) according to the International System of Units.

### Statistical analysis

Numerical variables were defined by the mean or median values, and the normality of the data distribution was assessed using the Kolmogorov-Smirnov test. To analyze the relationship between categorical and numerical variables, Student’s T-test or Mann-Whitney U test (for independent groups) and paired T-test or Wilcoxon test (for dependent groups) were used. Correlation analysis was performed using either the Pearson’s or Spearman’s test, as required. A *p*-value of less than 0.05 was considered statistically significant. The software SPSS v20.0 (SPSS Inc., Chicago, IL) was used for the statistical analysis.

## Results

**Fig. 1:**
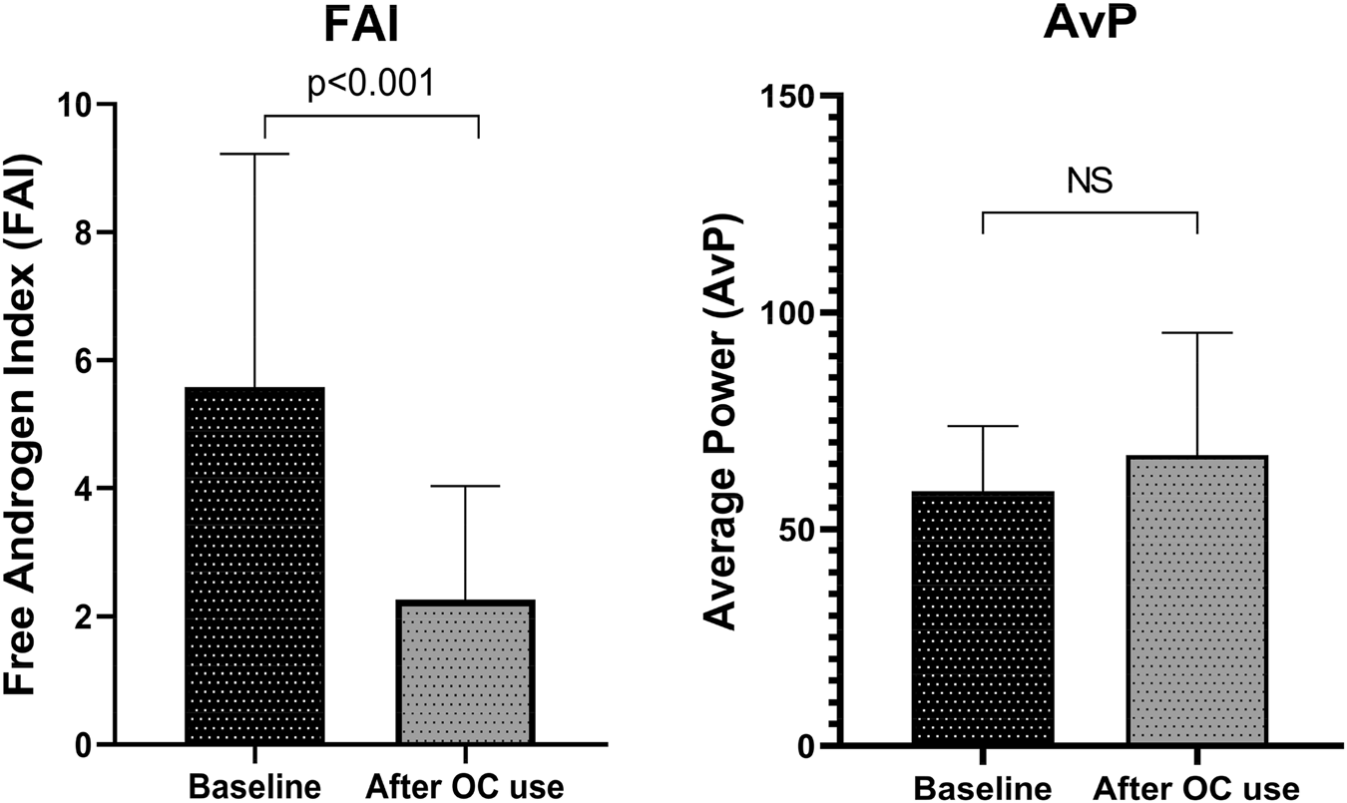
Changes in FAI and AVP in women with PCOS following oral contraceptive use

Total testosterone, FAI, and HOMA-IR were higher in patients with PCOS than healthy controls at baseline (*p* < 0.001, *p* = 0.001, *p* = 0.004, respectively). Among PCOS patients, 80% had phenotype A, and 20% had phenotype B. The median duration of OC use in the PCOS group was 121 days (IQR: 85–149). Testosterone levels and FAI showed significant reduction following OC use (*p* = 0.02, *p* < 0.001, respectively) (Table 1), (Fig. 1).

**Table 1:** Clinical, hormonal and metabolic assessments in women with PCOS and healthy controls at baseline and after OC use

Clinics	Control *N* = 20 SD/IQR	PCOS *N* = 20 SD/IQR	PCOS (3 months) *N* = 20 SD/IQR	*P* value (Control vs PCOS)	*P* value (PCOS pre to post)
Age	22 (20–24.7)	20.5 (20–22.7)		0.21	
BMI	24.3 (21.3–25.9)	26.1 (22.6–28.2)	25.9 (22.3–28)	0.21	0.14
WHR	0.8 (±0.05)	0.8 (±0.05)	0.8 (±0.06)	0.89	0.36
Testosterone	25.7 (±6.2)	53.5 (±16.4)	44.2 (±13.6)	<0.001	0.02
FAI	1.9 (1.5–2.5)	5.1 (2.4–7.9)	1.8 (0.8–2.9)	0.001	<0.001
Glucose	86.5 (±5.1)	85.1 (±7.7)	83.2 (±6.1)	0.50	0.27
Insulin	6.1 (3.9–7.8)	9.9 (6.8–12.1)	8.8 (5.9–13.1)	0.002	0.46
HOMA-IR	1.2 (0.8–1.7)	2.2 (1.3–2.6)	1.9 (1.2–2.7)	0.004	0.53
Total Cholesterol	180.6 (159.8–208.4)	187.0 (166.9–196)	190.2 (180.8–227.9)	0.79	0.02
LDL	108 (93.3–135)	110 (102.5–130.8)	112 (101–139.5)	0.86	0.13
HDL	53.8 (±11.7)	52 (±11.1)	56.7 (±14.3)	0.61	0.09
Trigliserid	66.5 (51.3–100)	74.5 (53.3–100.3)	96 (72.8–146)	0.52	0.04

Data are given as mean (SD) and median (IQR) where appropriate

*BMI* body mass index, *FAI* free androgen ındex, *HDL* high-density lipoprotein, *HOMA-IR* homeostasis model assessment index-insulin resistance, *LDL* low-density lipoprotein, *TG* triglyceride, *WHR* waist to hip ratio

At baseline, paraspinal muscle mass and fat percentage were comparable between the PCOS and control groups. Similarly, no significant differences were observed in baseline measurements of thigh muscle mass and fat fraction. Following OC use, no significant changes were observed in the composition assessments of the PCOS group (Table 2).

**Table 2:** Body composition analyses of women with PCOS and controls

Clinics	Control *N* = 20 SD/IQR	PCOS *N* = 20 SD/IQR	PCOS (3 months) *N* = 20 SD/IQR	*P* value (Control vs PCOS)	*P* value (PCOS pre to post)
Vertebral Fat %	28.8 (24.1–33.7)	30.9 (24.9–38.9)	33 (27.2–38.6)	0.52	0.97
Paraspinal Fat %	5.6 (4–7)	6.9 (4.8–8.3)	6.4 (5.3–8.2)	0.16	0.65
Paraspinal Mass	43.3 (±9)	42.8 (±9)	44 (±8.4)	0.85	0.15
Thigh Fat %	6.3 (±2.3)	6.9 (±2)	7.1 (±1.9)	0.40	0.66
Thigh Mass	108.6 (±15.6)	108.7 (±16.9)	106.7 (±14.9)	0.99	0.17

Data are given as mean (SD) and median (IQR) where appropriate

The PCOS group exhibited significantly greater AvP of the knee extensors at 60°/sec compared to controls (*p* = 0.002). There was no significant difference between the two groups in total work and peak torque/body weight (PTQ/BW) measurements (Table 3). Baseline analyses revealed positive correlations between AvP and total testosterone and FAI levels across the entire study population (r = 0.45, *p* = 0.004, r = 0.318, *p* = 0.045, respectively). No statistically significant change in AvP values was observed in the PCOS group following OC use (Table 3), (Fig. 1).

**Table 3:** Muscle strength analyses of women with PCOS and controls

Clinics	Control *N* = 20 SD/IQR	PCOS *N* = 20 SD/IQR	PCOS (3 months) *N* = 20 SD/IQR	*P* value (Control vs PCOS)	*P* value (PCOS pre to post)
Extensor /60					
T work (J)	268.4 (±71.8)	262.4 (±69.1)	301.1 (45.5–404.3)	0.79	0.47
AvP (W)	46.7 (40.3–51.1)	54.5 (48.9–70)	69.5 (48.7–79.9)	0.002	0.17
PTQ/BW (%)	168.5 (±36)	161.3 (±39.9)	162.6 (±60.1)	0.55	0.92
PTQ	101.9 (±17.9)	99.6 (±19.2)	113.6 (86.9–130.7)	0.70	0.39
Flexor /60					
T work (J)	109.7 (88–174)	98.1 (84.8–129.1)	132.7 (98.9–201.1)	0.21	0.03
AvP (W)	22.1 (±10.5)	23.9 (±11.3)	28.8 (±13.9)	0.60	0.15
PTQ/BW (%)	70 (±24.6)	61.3 (±20.8)	69.6 (62.8–84.3)	0.23	0.11
PTQ	40.9 (34.6–55.6)	35.4 (32–43)	43.5 (34.4–59.1)	0.19	0.10

Data are given as mean (SD) and median (IQR) where appropriate

*AvP* average power, *PTQ* peak torque, *PTQ/BW* peak torque/body weight, *T.Work* total work

## Discussion

This study demonstrated that short-term OC use alleviates androgen excess but does not impact muscle composition or strength in women with PCOS. Despite a reduction in androgen levels following OC use, no statistically significant changes were observed in AvP levels, which were initially higher in women with PCOS compared to healthy controls, or in body composition.

PCOS is characterized by hyperandrogenism and exhibits a significant positive correlation between serum androgen levels and muscle mass [13]. Androgen-driven anabolic reactions may explain the observed increase in muscle mass in women with PCOS, as evidenced by cross-sectional studies reporting greater trunk muscle mass, handgrip strength, and appendicular lean mass compared to BMI-matched controls [28, 29]. However, the relationship between hyperandrogenism and muscle mass in PCOS is complex and not uniformly observed across all studies. In the present study, despite higher androgen levels in women with PCOS, no significant differences in body composition were observed compared to healthy controls. Participants in our study had BMIs ranging from 18–35 kg/m^2^ representing normal weight, overweight and class I obese individuals. While obesity was not excluded, the study population lacked individuals with more severe obesity (BMI ≥ 35 kg/m^2^), which may have reduced variability in muscle mass and quality due to excess adiposity. According to recent meta-analyses, rather than androgen excess or insulin resistance, the higher lean mass in PCOS is primarily due to the higher rates of overweight and obesity in this population [30]. These outcomes suggest that, although hyperandrogenism and insulin resistance are central to PCOS pathophysiology, they may not consistently lead to measurable alterations in body composition.

Evidence from studies assessing how PCOS might affect muscular mass and strength remains inconclusive [30]. This inconsistency likely arises from heterogeneity of study populations regarding age, BMI and PCOS phenotypes and the confounding effects of insulin resistance and adiposity on muscle function [31, 32]. Previously studying 44 young women with PCOS and 32 age- and BMI-matched healthy controls, we have found increased lower extremity muscle strength in PCOS by isokinetic dynamometry that was positively correlated with androgen excess [33]. In a recent study of 34 aging women with PCOS and 32 age- and BMI-matched controls assessing musculoskeletal composition by dual-energy x-ray absorptiometry (DEXA) and MRI-PDFF and muscle strength by isokinetic dynamometer, we reported similar muscular composition and function in women with PCOS compared to controls in late reproductive years [27]. These findings imply that age-related metabolic alterations may affect muscle function and composition outcomes in PCOS. The baseline findings of the current study are consistent with previous research and indicate that young women with PCOS exhibit higher muscle strength despite having comparable body composition to healthy controls. The relatively restricted BMI range (18–35 kg/m^2^) may have limited the detection of adiposity-related differences in muscle parameters, particularly in the absence of individuals with more severe obesity.

Limited research on the effects of OC use on body composition in women with PCOS has yielded conflicting results. A previous study found that an increase in fat percentage following OC therapy, as measured by bioelectrical impedance, despite no significant changes in weight or anthropometric measures [34]. Another study using DEXA found that administration of OC alone resulted in weight gain, which was evenly distributed across both upper and lower body regions. Furthermore, no correlation was observed between alterations in testosterone levels and shifts in fat distribution, suggesting that OC treatment for patients with PCOS did not improve fat distribution [35]. To our knowledge, this is the first study to investigate the relationship between OC use and muscle mass and strength in women with PCOS using gold-standard imaging and strength measurement tools. Although elevated testosterone levels have been associated with increased muscle mass and enhanced performance in female athletes [36], previous studies have shown that oral contraceptive use does not significantly influence muscle strength or body composition in athletes and the general population [37, 38]. Consistent with previous research on athletes and the general population, our findings suggest that OC use does not impact body composition or muscle strength also in women with PCOS.

Several limitations should be acknowledged. First, the sample size is relatively small, which may limit the statistical power to detect subtle changes in muscle parameters. Second, the follow-up duration may not be sufficient to observe long-term effects of OC use on muscle mass and strength. Third, muscle strength assessment was limited to the lower limbs, and upper limb or total body strength was not evaluated. Fourth, only PCOS phenotype A and B were included, which may restrict the generalizability of the findings to all PCOS phenotypes. Finally, potential confounding factors such as physical activity level and dietary intake were not systematically recorded, which could influence the outcomes. On the other hand, using MRI-PDFF and isokinetic dynamometry, valid and gold standard methods for the assessment of body composition and muscle function are among the strengths of the current study.

In conclusion, our findings indicate that short-term OC therapy ameliorates androgen excess in women with PCOS but does not lead to significant changes in muscle composition or strength as assessed by MRI-PDFF and isokinetic dynamometry. These findings suggest that musculoskeletal parameters may remain stable during short-term OC use. However, due to the study’s limited sample size, short follow-up duration, and focus on specific PCOS phenotypes, caution is warranted when generalizing these results. Future research with larger, more diverse populations and longer-term follow-up is essential to better understand the musculoskeletal implications of hormonal therapies in women with PCOS.

## Data Availability

The datasets collected and analyzed during this study are available upon reasonable request to the corresponding author.
